# The dichotomy of progress: Public perceptions of global health trends and antibiotic knowledge in Georgia

**DOI:** 10.1371/journal.pgph.0005037

**Published:** 2026-08-24

**Authors:** Zurab Alkhanishvili

**Affiliations:** Clinical and Translational Medicine Program, Faculty of Medicine, Ivane Javakhishvili Tbilisi State University, Tbilisi, Georgia; PLOS: Public Library of Science, UNITED STATES OF AMERICA

## Abstract

Substantial improvements in global population health have been documented over the past century; however, public perceptions of these long-term trends may not reflect empirical evidence. Misalignment between scientific progress and public understanding can affect trust, policy compliance, and the effectiveness of health governance. A nationally representative cross-sectional survey was conducted in Georgia between May and July 2023 among adults aged 18 years and older. Data were collected through face-to-face interviews using a structured questionnaire. The survey assessed public perceptions of long-term trends in global population growth, life expectancy, and under-five child mortality, as well as knowledge regarding antibiotic effectiveness against viral infections. Descriptive analyses and chi-square tests were applied to examine response patterns across sociodemographic groups. Among 1,876 respondents, recognition of declining child mortality was high (98.0%). In contrast, substantially lower proportions correctly identified increases in global population size (36.0%) and life expectancy (18.0%). Knowledge regarding antibiotic effectiveness was limited, with 32.0% of respondents correctly indicating that antibiotics are ineffective against viral infections. Variation in correct responses was observed across age groups. The findings reveal a persistent gap between documented global health progress and public perceptions in Georgia. While highly visible health indicators appear to be better recognized, broader historical trends and clinically relevant knowledge remain poorly understood. Addressing this progress - perception gap may represent an important component of strengthening public trust and improving the effectiveness of future public health governance.

## 1. Introduction

The COVID-19 pandemic reaffirmed that infectious disease outbreaks remain a persistent challenge to population health and continue to test the resilience of national and global health systems [1,2]. Throughout the past century, major public health crises have shaped the evolution of health governance and contributed to substantial improvements in population health outcomes, including increased life expectancy and reductions in preventable mortality [2,3].

Despite these measurable advances, recent global health emergencies have revealed persistent weaknesses in public engagement with scientific evidence [4–6]. During the COVID-19 pandemic, scientific progress was accompanied by widespread uncertainty, skepticism, and misunderstanding, which limited adherence to public health recommendations in many settings [4–7]. These patterns highlight that technical capacity alone is insufficient for effective health governance if public perception does not align with established evidence [2,4].

Beyond pandemic contexts, discrepancies between documented health progress and public belief have become increasingly visible in public discourse [5,6]. Perceptions that health conditions are deteriorating, that previous generations experienced better health, or that medical interventions are becoming less effective frequently persist despite long-term improvements documented by demographic and epidemiological data [3,5]. Such misalignments have implications not only for individual decision-making but also for institutional legitimacy, policy compliance, and long-term support for public health systems [5–8].

### 1.1 Theoretical framework: The progress–perception gap

Existing research has extensively examined determinants of public engagement with health information, including health literacy, misinformation, institutional trust, and risk perception [4–8]. These frameworks have contributed valuable insights into how individuals acquire and interpret health-related information and how inaccurate claims circulate within societies [4,6–8]. However, they offer more limited explanation of how populations evaluate long-term historical trajectories of health progress.

Public beliefs regarding whether life expectancy has increased, child mortality has declined, or global population growth has occurred represent broader interpretative judgments rather than discrete knowledge items. Such judgments may be shaped by collective memory, cultural narratives, and lived experience, and may persist even when scientific data are accessible [5,6]. As a result, population-level perceptions of historical change may diverge systematically from empirical evidence [3,5].

In this study, this divergence is conceptualized as the progress–perception gap, defined as the misalignment between objectively documented population-level health improvements and collective public beliefs regarding long-term health trajectories [5,6]. Unlike traditional health literacy models, which focus on factual comprehension, the progress–perception gap captures a structural disconnect between measurable outcomes and societal interpretation of progress.

Unlike misinformation-centered frameworks, which address the veracity of specific claims, the progress–perception gap concerns broader societal evaluations of health directionality over time - evaluations that may not be readily corrected through information provision alone, and whose persistence may erode public confidence in health institutions and undermine long-term governance effectiveness [2,4–8].

### 1.2 Study aim and objectives

Within this theoretical framework, the present study aims to quantify the extent of the progress–perception gap among the adult population of Georgia using data from a nationally representative survey conducted in 2023. The study examines public perceptions regarding long-term trends in global population growth, life expectancy, and child mortality, and compares these perceptions with established demographic and epidemiological evidence [3].

In addition to historical population indicators, the study includes a practical clinical knowledge item related to antibiotic efficacy. This component was incorporated to assess whether misalignment between scientific evidence and public belief extends beyond abstract population-level trends to domains with direct relevance for individual health behavior. Misconceptions regarding antibiotic use represent a well-documented global challenge, particularly in the context of viral respiratory infections, where incorrect beliefs may contribute to inappropriate treatment practices and antimicrobial resistance [9,10]. Even in high-income settings such as the United States, a substantial share of the public incorrectly believes that antibiotics are effective against viral infections, underscoring that this is not a context-specific problem [11].

By integrating both historical demographic indicators and a clinically relevant knowledge domain, the study seeks to examine whether the progress–perception gap reflects a broader structural pattern of public understanding rather than isolated informational deficits.

## 2. Materials and methods

### 2.1 Study design and setting

This study employed a cross-sectional, population-based survey design conducted in Georgia between May and July 2023. The survey targeted the adult population aged 18 years and older and aimed to assess public perceptions regarding long-term global health trends and selected aspects of medical knowledge. Data were collected through face-to-face interviews using a structured, paper-based questionnaire. Fieldwork was conducted across all 11 administrative regions of Georgia, excluding the occupied territories. To ensure geographic representativeness, the population was stratified by region, with each region treated as a primary stratum.

### 2.2 Sampling and participants

A multistage stratified sampling approach was applied. In the first stage, primary sampling units (PSUs) were defined based on regional strata. Within each region, secondary sampling units (SSUs) were defined and households were selected within each SSU using the random walk method [12]. This method was selected to ensure random coverage given the absence of a comprehensive and up-to-date national household address registry, particularly in rural settlements. Within selected households, one eligible respondent aged 18 years or older was chosen according to the “last birthday” method. A total of 1,876 adult participants completed the survey and were included in the final analysis. Of the 1,921 individuals approached, 1,876 agreed to participate, yielding a response rate of 97.7%. The achieved sample size provided an estimated margin of error of approximately ±2.5% at the 95% confidence level.

### 2.3 Data collection procedures

Data collection was conducted by the principal investigator using a standardized interview protocol to ensure consistency in questionnaire administration and minimize procedural variability. All interviews were conducted face-to-face in Georgian using a structured, paper-based questionnaire. Participation was voluntary, and no financial incentives were provided.

### 2.4 Survey instrument and variables

This study employed a structured questionnaire developed to assess public perceptions of long-term global health trends and selected aspects of medical knowledge (see S1 File for the complete survey instrument). The instrument was designed specifically for this study and administered in Georgian. The questionnaire consisted of six sections. The first section captured geographic stratification variables, including region and settlement type (urban or rural). The second section included a standardized introduction and verbal informed consent procedure. Sociodemographic characteristics collected included gender, age, and highest completed level of education. Perceptions of global health trends were assessed using three core items addressing respondents’ beliefs regarding changes over the past 100 years in (1) global population size, (2) average life expectancy, and (3) under-five child mortality. For each item, respondents selected one response option indicating whether they believed the indicator had increased, decreased, or remained approximately the same, with an additional option for “don’t know/refused.” Medical knowledge was assessed through a single true–false statement evaluating understanding of antibiotic efficacy against viral infections.

Survey items **followed the fact-based, multiple-choice format used in international studies of public misperceptions of long-term global development trends** [13], and the antibiotic item followed the formulation used in international antimicrobial resistance awareness surveys [14]. The Georgian-language questionnaire was pre-tested on 15 adults, after which minor wording adjustments were made. As the instrument comprised discrete objective knowledge items rather than a latent scale, internal-consistency reliability metrics were not applicable.

Each item was scored dichotomously. For the perception items, responses matching the direction established by demographic and epidemiological evidence (increase in global population, increase in life expectancy, decline in under-five mortality) were coded as correct, and the antibiotic item was coded as correct when respondents indicated that antibiotics are ineffective against viral infections. Incorrect answers and “don’t know/refused” responses were combined into a single misconception/uncertainty category, consistent with Table 1.

**Table 1:** Public perceptions of selected global health indicators compared with scientific evidence.

Health indicator	Survey question topic	Scientific evidence	Correct response % (95% CI)	Misconception / uncertainty % (95% CI)
Global population	Increased or decreased over the past 100 years?	Increased	36.0 (33.8–38.2)	64.0 (61.8–66.2)
Life expectancy	Increased or decreased over the past 100 years?	Increased	18.0 (16.3–19.7)	82.0 (80.3–83.7)
Child mortality	Increased or decreased over the past 100 years?	Decreased	98.0 (97.4–98.6)	2.0 (1.4–2.6)
Antibiotic efficacy	Are antibiotics effective against viral infections (e.g., common cold, influenza, COVID-19)?	False (ineffective)	32.0 (29.9–34.1)	68.0 (65.9–70.1)

**Note:** Misconception/uncertainty category includes incorrect responses and “don’t know/refused” answers. Percentages are unweighted proportions of all 1,876 respondents.

### 2.5 Statistical analysis

Descriptive statistics were used to summarize respondents’ demographic characteristics and distribution of responses, including frequencies and percentages. Prevalence estimates are reported as unweighted proportions. Inferential analyses were conducted to explore associations between selected sociodemographic variables and perception outcomes using chi-square tests. 95% Confidence Intervals (CIs) were calculated for key prevalence estimates to assess precision. Statistical analyses were performed using IBM SPSS Statistics (version 29.0.1.1). A p-value threshold of <0.05 was used for inferential testing. Given the study’s exploratory objective, inferential findings were interpreted cautiously and primarily used to support descriptive patterns. Post-stratification weighting to Geostat targets for gender, age, and region was applied as a sensitivity analysis and changed all estimates by less than one percentage point (see deposited file). Subgroup comparisons and chi-square tests are reported unweighted.

### 2.6 Ethical considerations

The study protocol was reviewed and approved by the Institutional Review Board (IRB) of the Health Research Union (IRB# 2023-00) on January 16, 2023. The Board determined that the study provided adequate safeguards for the rights and welfare of the participants. The study was conducted in accordance with the principles of the Declaration of Helsinki. All potential participants were provided with standardized information regarding the study objectives, procedures, and voluntary nature of participation. Verbal informed consent was obtained from all participants prior to the interview. The consent process was documented by the interviewer on the questionnaire form in accordance with IRB-approved procedures. This procedure was approved by the IRB to maintain respondent anonymity. Participation was fully voluntary, and respondents were informed of their right to decline participation or withdraw at any time without consequences. The survey was conducted anonymously, no personally identifiable information was collected, and all data were stored securely and analyzed in aggregated form to ensure confidentiality.

## 3. Results

### 3.1 Sociodemographic characteristics

A total of 1,876 respondents were included in the final analysis. The sample was predominantly female (66%) and largely composed of residents of rural settlements (78%). The mean age of participants was 47 years. The survey covered all 11 administrative regions of Georgia, excluding the occupied territories.

All estimates presented below are unweighted proportions calculated from the achieved sample of 1,876 respondents.

### 3.2 Perceptions of long-term demographic trends

Public understanding of long-term global demographic trends was limited across several indicators.

Regarding global population growth, 36.0% (95% CI: 33.8–38.2) of respondents correctly identified that the world population has increased over the past century. Correct responses did not differ significantly by gender (36.2% among women and 35.6% among men; χ² = 0.04, df = 1, p = 0.83).

Awareness of trends in life expectancy was notably lower. Only 18.0% (95% CI: 16.3–19.7) of respondents correctly recognized that average global life expectancy has increased over the past 100 years. As with population growth, no significant gender difference was observed (17.3% among women and 19.4% among men; χ² = 1.18, df = 1, p = 0.28).

Perceptions regarding child mortality showed considerably higher accuracy. The vast majority of respondents (98.0%) correctly identified that global under-five mortality has declined compared to one hundred years ago (Table 1).

### 3.3 Knowledge regarding antibiotic effectiveness

Knowledge related to antibiotic efficacy revealed substantial gaps within the study population. When asked whether antibiotics are effective in treating viral infections such as the common cold, influenza, or COVID-19, only 32.0% (95% CI: 29.9–34.1) of respondents correctly stated that antibiotics are ineffective against viral illnesses. The remaining 68.0% either incorrectly believed antibiotics to be effective or reported uncertainty.

Differences in correct responses were observed across age groups. The highest proportion of correct knowledge was recorded among respondents aged 18–29 years (47.3%), followed by respondents aged 60 years and older (30.0%), while those aged 30–59 years demonstrated the lowest proportion of correct responses (29.0%). An association between age group and correct knowledge regarding antibiotic use was observed (χ² = 35.9, df = 2, p < 0.001), with a small effect size (Cramer’s V = 0.14).

Respondents living in urban and rural settlements demonstrated similar levels of correct antibiotic knowledge (32.7% and 31.8%, respectively). This association was not statistically significant (χ² = 0.08, df = 1, p = 0.77). Correct antibiotic knowledge also did not differ significantly by educational attainment (primary 35.1%, secondary 32.8%, vocational 31.7%, higher 30.0%; χ² = 1.9, df = 3, p = 0.60), with 38 respondents who declined to report education excluded from this comparison.

## 4. Discussion

Our 2023 nationally based survey in Georgia indicates a consistent mismatch between documented long-term global health trajectories and public perceptions of those trajectories. While the decline in under-five mortality was correctly recognized by most respondents, markedly lower accuracy was observed for world population growth, global life expectancy trends, and antibiotic effectiveness. This pattern suggests that public understanding may be more aligned with highly visible or frequently communicated indicators, while broader, time-comparative judgments about progress remain less anchored in evidence [3,15].

This misalignment is consequential for health governance because public cooperation during crises depends not only on technical capacity but also on shared understanding of risk and the credibility of public recommendations. During COVID-19, many settings experienced a parallel challenge of rapid scientific progress alongside uncertainty and contested information environments, which complicated population-level adherence to public health measures and contributed to inconsistent responses across contexts [4,16,17]. In such conditions, governance effectiveness is partly mediated by how the public interprets evidence, not only by whether evidence exists.

A further interpretation is that public health can, in part, become vulnerable to its own success. Over decades, sustained reductions in infectious disease mortality and visible improvements in survival have reduced the everyday salience of some threats for many communities. When major hazards feel distant, attention to scientific evidence and preventive norms may weaken, creating space for simplified narratives, selective memories, and episodic crisis-driven perceptions rather than time-grounded assessments of progress. This dynamic may be amplified in environments where competing information flows are intense and where institutional messaging is fragmented across many actors and platforms [18,19]. In practical terms, long-run gains can become “background noise,” while shocks, controversies, and personal anecdotes become disproportionately influential in how progress is judged.

The antibiotic item in our survey illustrates how evidence–perception misalignment can translate into practical misconceptions with potential downstream consequences. Misunderstanding antibiotic effectiveness for viral infections remains widely documented in community surveys internationally and has been repeatedly highlighted as a challenge for antimicrobial stewardship, particularly when respiratory viral illness dominates public attention [20,21]. In our data, correct understanding varied by age group, indicating that information environments and access patterns may matter for how medical knowledge is formed and maintained. Given the broader global burden associated with antimicrobial resistance, persistent misconceptions in the general public are relevant not only as an educational gap but also as a governance and system-sustainability issue [9].

Despite global awareness campaigns and long-standing efforts to promote rational antibiotic use, misconceptions regarding antibiotic efficacy remain widespread across diverse populations. Systematic reviews and large-scale surveys indicate that fewer than half of adults correctly understand that antibiotics are ineffective against viral infections such as the common cold or influenza [20,21]. Similar patterns have been documented across European and other high-income settings, where substantial proportions of the population continue to associate antibiotics with faster recovery from viral illnesses [22]. These persistent misunderstandings represent a global phenomenon rather than a context-specific failure and contribute directly to inappropriate antibiotic consumption and the accelerating threat of antimicrobial resistance, which is recognized as one of the most critical global public health challenges by international health authorities [9].

A comparison with international evidence helps situate these findings. In a nationally representative United States survey, 45 percent of adults correctly recognized that antibiotics are ineffective against viral infections [11], and a peer-reviewed United States survey found that more than 40 percent of adults still considered antibiotics appropriate for symptoms typical of viral illness [23]. A recent systematic review and meta-analysis of 227 studies from 98 countries similarly reported that only 42.1 percent (95% CI 39.2–44.9) of the general public correctly recognized that antibiotics are ineffective against viral infections [24]. The correct-response proportion in Georgia (32.0 percent) was therefore lower than both the United States figure and the pooled global estimate, indicating a wider knowledge gap, although misconceptions persist across all these settings despite sustained awareness efforts. This supports the interpretation that misunderstanding of antibiotic efficacy against viral infections is a global phenomenon rather than a failure specific to any single health system.

A key implication for policy and practice is that narrowing the progress–perception gap likely requires more than one-way information provision. During COVID-19, the literature consistently points to the importance of trust, coherent messaging, and communication strategies that fit the platforms and interpretive habits of target groups, including digital channels [4,16,17]. In fragmented governance environments, multiple credible messengers (public institutions, professional groups, community organizations) can be an asset only if coordination improves clarity rather than multiplying conflicting signals [17,19]. For Georgia specifically, where this study documents substantial misunderstanding of some foundational global indicators, strategies that connect abstract trends to locally meaningful narratives and trusted community-level intermediaries may be more plausible than purely technical fact campaigns.

### 4.1 Study limitations

This study should be interpreted in light of several limitations. First, the survey captured public perceptions at a single point in time, which limits inference about temporal changes or causal relationships. Second, the questionnaire was intentionally concise and focused on a limited number of indicators, which constrained the depth of measurement. Third, the achieved sample over-represented women and rural residents relative to the national population; although post-stratification weighting applied as a sensitivity analysis produced closely similar estimates, residual bias cannot be fully excluded.

In addition, the study relied exclusively on quantitative self-reported data and did not include a qualitative component, which restricts insight into the underlying narratives, experiences, and information pathways shaping public perceptions. Future research would benefit from mixed-method designs and cross-national comparisons to further examine the mechanisms sustaining the progress - perception gap.

## 5. Conclusion

This study demonstrates a substantial gap between documented long-term global health progress and public perceptions of that progress among the adult population of Georgia. While declines in under-five mortality were widely recognized, misconceptions regarding population growth, life expectancy trends, and antibiotic effectiveness remained prevalent.

These findings highlight that public health performance cannot be assessed solely through scientific and institutional achievements. Societal understanding of progress plays a critical role in shaping trust, policy compliance, and long-term system sustainability. The progress–perception gap identified in this study suggests that effective health governance requires not only technical capacity but also sustained public engagement capable of translating historical and scientific evidence into meaningful societal understanding.

Addressing this gap represents an important priority for strengthening public trust and resilience in future health emergencies.

## Supporting information

S1 File Survey questionnaire. English translation of the structured questionnaire used for data collection.
(PDF)
