# Supplementary material for: The dichotomy of progress: Public perceptions of global health trends and antibiotic knowledge in Georgia
Source: PLOS Glob Public Health. 2026 Aug 24;6(8):e0005037. doi: 10.1371/journal.pgph.0005037 (PMC13502681; doi:10.1371/journal.pgph.0005037)
Supplement: Supplementary file 1 [file pgph.0005037.s001.pdf]

S1 File. Survey Questionnaire

Public Perceptions of Long-Term Global Health Trends and Selected Aspects of Medical Knowledge

Introduction

Hello. My name is ___________.

I am conducting a research study on public perceptions of long-term global health trends and selected aspects of medical knowledge.

Your participation in this study is entirely voluntary. The interview is anonymous, and no personally identifiable information will be collected. You may refuse to answer any question or stop the interview at any time without any consequences.

The interview will take approximately 5 minutes.

Do you agree to participate in this survey?

☐ Yes → proceed with the interview
☐ No → terminate the interview

Section 1. Geographic Information

1. **Region of residence**

☐ Tbilisi
☐ Adjara
☐ Guria
☐ Samegrelo–Zemo Svaneti
☐ Imereti
☐ Racha–Lechkhumi and Kvemo Svaneti
☐ Samtskhe–Javakheti
☐ Shida Kartli
☐ Kvemo Kartli
☐ Mtskheta–Mtianeti
☐ Kakheti

2. **Type of settlement**

☐ Urban
☐ Rural

Section 2. Sociodemographic Characteristics

3. **Gender**

☐ Female
☐ Male
☐ Prefer not to say

4. **Age (in completed years)**

☐ 18–29
☐ 30–44
☐ 45–59
☐ ≥60

5. **Highest completed level of education**

☐ Primary education
☐ Secondary education
☐ Vocational education
☐ Higher education (Bachelor’s degree or higher)
☐ Prefer not to say

Section 3. Perceptions of Long-Term Global Health Trends

6. **In your opinion, over the past 100 years, has the world population:**

☐ Increased
☐ Decreased
☐ Remained approximately the same
☐ Don’t know / difficult to answer

7. **In your opinion, over the past 100 years, has average life expectancy worldwide:**

☐ Increased
☐ Decreased
☐ Remained approximately the same
☐ Don’t know / difficult to answer

8. **In your opinion, over the past 100 years, has mortality among children under five years of age worldwide:**

☐ Increased
☐ Decreased

☐ Remained approximately the same
☐ Don't know / difficult to answer

Section 4. Medical Knowledge

9. **Antibiotics are effective for treating viral infections such as the common cold, influenza, or COVID-19.**

☐ True
☐ False
☐ Don't know

Section 5. Interviewer Documentation

10. **Interview outcome**

☐ Completed
☐ Partially completed
☐ Refused

11. **Date of interview**

___ / ___ / 2023

Thank you very much for your participation.

Notes for Reviewers

This questionnaire was developed specifically for the present study to assess public perceptions of long-term global health trends and selected aspects of medical knowledge. All questions were administered orally in Georgian using a standardized face-to-face interview protocol. The version presented here represents the translated questionnaire corresponding to the instrument used during data collection.
