# Supplementary material for: The dichotomy of progress: Public perceptions of global health trends and antibiotic knowledge in Georgia
Source: PLOS Glob Public Health. 2026 Aug 24;6(8):e0005037. doi: 10.1371/journal.pgph.0005037 (PMC13502681; doi:10.1371/journal.pgph.0005037)
Supplement: Supplementary file 2 [file pgph.0005037.s004.pdf]

Dear Editor and Reviewers,

Thank you for the opportunity to revise the manuscript entitled *"The Dichotomy of Progress: Public Perceptions of Global Health Trends and Antibiotic Knowledge in Georgia"* (Manuscript ID: PGPH-D-25-02074R1).

I appreciate the reviewer's specific feedback aimed at improving the clarity and conciseness of the text. I have carefully addressed all comments in this revised version. Below is the point-by-point response detailing the modifications made to the manuscript.

Response to Reviewer 1

Comment 1: The introduction section and the theoretical framework are somewhat long and generic. Please condense these sections to make the manuscript more focused.

Response: The opening discussion regarding the historical context of health crises was condensed into a single sentence, while retaining the foundational citations.

- Overlapping sentences concerning COVID-19 uncertainty and public compliance were removed to improve the narrative flow.
- In the Theoretical Framework, two lengthy paragraphs were replaced with a single paragraph that directly contrasts the "progress–perception gap" with traditional misinformation-centered approaches, maintaining all relevant citations [2,4–7,10].
- These modifications have reduced the volume of the introductory framework without compromising its conceptual foundation.

Comment 2: The description of the "Random Walk" method needs further elaboration to clarify how household selection was performed in the field, along with an appropriate methodological reference.

Response: The description of the sampling method in the text was updated and the appropriate methodological reference was added, as recommended by the reviewer:

- Bauer JJ. Selection errors of random route samples. Sociological Methods & Research. 2014;43(3):519–544. doi:10.1177/0049124114521150.

Comment 3: To comply with open science practices and the journal's strict policies, please ensure that the minimal underlying dataset is deposited in a public repository.

Response: In accordance with the journal's data availability mandate, I have deposited the de-identified minimal underlying dataset, along with the corresponding survey codebook, in the Open Science Framework (OSF) repository. The data is publicly accessible without restriction via the following permanent URL: **https://osf.io/a29du**. The Data Availability Statement in the manuscript submission fields has been updated accordingly.

Additional Corrections by the Author

1. Correction of a technical typo in the Discussion section:

During a final quality review of the text, a minor technical error regarding the percentages was identified in Sentence 120 of the Discussion section. The wording has been corrected to accurately reflect that **32.0%** of respondents demonstrated correct knowledge regarding antibiotic efficacy against viral infections, ensuring perfect consistency with the values presented in the Results and Table 1.

Thank you once again for your time and guidance throughout the review process.

Sincerely,

Zurab Alkhanishvili

Corresponding Author
