# Supplementary material for: The dichotomy of progress: Public perceptions of global health trends and antibiotic knowledge in Georgia
Source: PLOS Glob Public Health. 2026 Aug 24;6(8):e0005037. doi: 10.1371/journal.pgph.0005037 (PMC13502681; doi:10.1371/journal.pgph.0005037)
Supplement: Supplementary file 3 [file pgph.0005037.s003.docx]

**Response to Reviewers**

**Manuscript ID:** PGPH-D-25-02074
**Title:** The Dichotomy of Progress: Public Perceptions of Global Health Trends and Antibiotic Knowledge in Georgia

**General response**

I thank the editor and reviewers for their detailed and constructive feedback. All comments were carefully considered and addressed. The scope of the requested revisions, including clarification of theoretical framing, strengthening of methodological transparency, and improvement of language clarity, necessitated substantial restructuring and partial rewriting of the manuscript. As a result, the revised version reflects comprehensive revisions across all major sections to ensure conceptual coherence, analytical consistency, and alignment with the journal’s standards.

**Reviewer #1**

**Comment: Clarification of the perception gap concept and manuscript structure.**

**Response:**
The Introduction was revised to clearly define the progress–perception gap as an analytical concept and to distinguish it from related constructs such as health literacy and misinformation. In response to this conceptual clarification and the associated language refinements, the manuscript required structural reorganization, with theoretical framing presented prior to the study objectives and empirical sections to improve overall logical flow.

**Comment: Use of post-Soviet or transitional context requires clearer justification.**

**Response:**
To avoid speculative contextual interpretation, explicit references to post-Soviet or transitional status were removed from the analytical framing of the study. The revised manuscript therefore focuses on empirically observable perception patterns rather than historically or politically interpretive explanations.

**Comment: Absence of a qualitative component.**

**Response:**
The absence of qualitative data is now explicitly acknowledged in the Study Limitations section. The manuscript also outlines the potential value of future mixed-methods research for examining the narratives and mechanisms underlying public perceptions.

**Reviewer #2**

**Comment: Insufficient theoretical framework.**

**Response:**
A dedicated theoretical framework section was added to conceptualize the progress–perception gap and to clearly distinguish it from existing models of health literacy, misinformation, and risk perception. The integration of this framework required corresponding revisions across the Introduction, Study Aim, and Discussion sections to ensure conceptual consistency.

**Comment: Survey instrument not adequately described.**

**Response:**
The Methods section was expanded to provide a detailed description of the survey instrument and measured variables. The full questionnaire is provided as Supporting Information (S1 File). As the survey was administered in Georgian, the version included in the Supporting Information represents the translated version of the original questionnaire used during data collection.

**Comment: Statistical analysis and reporting insufficiently detailed.**

**Response:**
Statistical reporting was clarified throughout the Methods and Results sections. Chi-square statistics, degrees of freedom, p-values, confidence intervals, and effect size measures (Cramer’s V, where applicable) are now reported to enhance transparency and interpretability of the findings.

**Comment: Overgeneralization of findings beyond the Georgian context.**

**Response:**
The Discussion was revised to limit extrapolation beyond the study setting. References to global health governance are now framed as interpretive implications rather than empirical generalizations, with explicit emphasis on the national scope of the data.

**Comment: Limitations section insufficient.**

**Response:**
The Study Limitations subsection was expanded to address the cross-sectional design, language-related constraints of the Georgian-language survey, the absence of qualitative data, and the non-analysis of education level despite its collection.

**Comment: Abstract lacks structure.**

**Response:**
The Abstract was rewritten to clearly present background, methods, principal findings, and conclusions in accordance with the journal’s structural requirements.

**Editorial requirement**

**Comment: Clarification of verbal informed consent procedures.**

**Response:**
The Ethics Statement was expanded to describe the procedure for obtaining verbal informed consent, including its documentation by the interviewer and its approval by the Institutional Review Board, in accordance with IRB-approved protocols.
