# Supplementary material for: The dichotomy of progress: Public perceptions of global health trends and antibiotic knowledge in Georgia
Source: PLOS Glob Public Health. 2026 Aug 24;6(8):e0005037. doi: 10.1371/journal.pgph.0005037 (PMC13502681; doi:10.1371/journal.pgph.0005037)
Supplement: Supplementary file 4 [file pgph.0005037.s005.docx]

**Response to the Editor**

**Manuscript ID: PGPH-D-25-02074R2**

Title: The Dichotomy of Progress: Public Perceptions of Global Health Trends and Antibiotic Knowledge in Georgia

Journal: PLOS Global Public Health

Dear Dr. Kokkinias,

Below is a point-by-point response to the three remaining comments. All changes are marked in the revised manuscript with tracked changes, and inserted text is shown here in indented italics.

**Editor comment 1: Validation of the survey instrument**

**Comment:** Please add additional information regarding how the survey instrument was validated.

**Response:** Section 2.4 now describes how content and face validity were established. The perception items follow the format of established international surveys of public knowledge of long-term demographic trends, and the antibiotic item follows the standardized formulation used in international antimicrobial resistance awareness surveys; both source instruments are now cited. The Georgian-language questionnaire was pre-tested with 15 adults, after which minor wording adjustments were made. We have also clarified that internal-consistency reliability metrics were not applicable, as the instrument consists of discrete objective knowledge items with a single correct response rather than a multi-item latent scale. Text added:

*Survey items were adapted from established international instruments, supporting content validity: the perception items followed the format used in international surveys of public knowledge of long-term demographic trends [21], and the antibiotic item followed the formulation used in international antimicrobial resistance awareness surveys [22]. The Georgian-language questionnaire was pre-tested on 15 adults, after which minor wording adjustments were made. As the instrument comprised discrete objective knowledge items rather than a latent scale, internal-consistency reliability metrics were not applicable.*

**Editor comment 2: Scoring of the perception items in the Methods**

**Comment:** A description of the perception scoring is included as a note beneath Table 1. Please also include how the perception items were scored in the Methods section.

**Response:** The scoring procedure has been added to section 2.4 and no longer appears only in the note to Table 1. Text added:

*Each item was scored dichotomously. For the perception items, responses matching the direction established by demographic and epidemiological evidence (increase in global population, increase in life expectancy, decline in under-five mortality) were coded as correct, and the antibiotic item was coded as correct when respondents indicated that antibiotics are ineffective against viral infections. Incorrect answers and “don’t know/refused” responses were combined into a single misconception/uncertainty category, consistent with Table 1.*

**Editor comment 3: Comparison with United States literature**

**Comment:** It would be beneficial to compare and contrast your results with the existing literature on antibiotic knowledge and perceptions in the United States within the Introduction and Discussion sections.

**Response:** A comparison with United States literature has been added to both sections, together with a recent global meta-analysis that places the Georgian result in international context. A sentence was added to the Introduction (section 1.2):

*Even in high-income settings such as the United States, a substantial share of the public incorrectly believes that antibiotics are effective against viral infections, underscoring that this is not a context-specific problem [20].*

The following passage was added to the Discussion, following the section on antibiotic knowledge:

*A comparison with international evidence helps situate these findings. In a nationally representative United States survey, 45 percent of adults correctly recognized that antibiotics are ineffective against viral infections [20], and a peer-reviewed United States survey found that more than 40 percent of adults still considered antibiotics appropriate for symptoms typical of viral illness [23]. A recent systematic review and meta-analysis of 227 studies from 98 countries similarly reported that only 42.1 percent (95% CI 39.2–44.9) of the general public correctly recognized that antibiotics are ineffective against viral infections [24]. The correct-response proportion in Georgia (32.0 percent) was therefore lower than both the United States figure and the pooled global estimate, indicating a wider knowledge gap, although misconceptions persist across all these settings despite sustained awareness efforts. This supports the interpretation that misunderstanding of antibiotic efficacy against viral infections is a global phenomenon rather than a failure specific to any single health system.*

**Additional corrections by the author**

While preparing the public dataset, we re-verified the subgroup analyses against the deposited respondent-level data and made the corrections listed below. **None of these affect the study’s conclusions, the primary prevalence estimates in Table 1, or the interpretation of the findings, and the Abstract is unchanged apart from the removal of one clause noted in point 2.**

**1. Gender comparisons (section 3.2).** The previously reported gender figures were compositional shares, that is, the proportion of all correct responses contributed by each gender, rather than the proportion of each gender answering correctly. Because women comprised 66% of the achieved sample, such shares reflect sample composition rather than knowledge. Both sentences now report within-group correct-response rates with formal tests, which show no significant gender difference for either item. Gender was not referred to in the Abstract, Discussion, or Conclusions, which are therefore unaffected.

**2. Settlement type (sections 3.3, Abstract, Discussion).** Exact proportions and the test statistic have been added for the urban and rural comparison (32.7% and 31.8%; p = 0.77). As the association was not significant, the passing reference to settlement type has been removed from one sentence in the Abstract and one in the Discussion, so that the text is consistent with the Results.

**3. Age group labels (section 3.3).** The analysis groups correspond to the survey age bands 18 to 29, 30 to 59, and 60 years and older. These were previously labelled 18 to 34, 35 to 54, and 55 years and older. The labels have been corrected and the proportions and test statistic recomputed from the deposited data. The direction and interpretation of the finding are unchanged: correct knowledge remains highest among the youngest group, and the association remains statistically significant with a small effect size.

**4. Educational attainment (sections 3.3 and 4.1).** Education was collected but not previously analysed. On analysis, correct antibiotic knowledge did not differ significantly by educational level (χ² = 1.9, df = 3, p = 0.60). This null finding has been added to section 3.3 and the corresponding limitation removed.

**5. Weighting (section 2.5).** For clarity we have specified that prevalence estimates are reported as unweighted proportions, and that post-stratification weighting to Geostat targets was applied as a sensitivity analysis, changing all estimates by less than one percentage point. Both the unweighted data and the weighting are available in the deposited file

**6. Editorial corrections.** A duplicated sentence in section 2.3 has been removed, and the Discussion heading, which was previously unnumbered, has been numbered so that the Conclusion is numbered 5 rather than 4.

**References**

In line with the journal requirement to verify the reference list, we identified two entries that appeared twice (van Bavel et al., Nature Human Behaviour 2020, and the Global Burden of Bacterial Antimicrobial Resistance analysis, Lancet 2022). The duplicates have been removed. The list has also been updated to accommodate the newly cited studies supporting the comparison requested above and the source instruments referred to in section 2.4. It now contains 24 entries and citations have been renumbered accordingly.

We hope the revised manuscript is now suitable for publication.

Sincerely,

Zurab Alkhanishvili

*Corresponding Author*
