# Supplementary material for: The dichotomy of progress: Public perceptions of global health trends and antibiotic knowledge in Georgia
Source: PLOS Glob Public Health. 2026 Aug 24;6(8):e0005037. doi: 10.1371/journal.pgph.0005037 (PMC13502681; doi:10.1371/journal.pgph.0005037)
Supplement: Peer review history

# Peer review: Decision Letter 0

**DOI:** 10.1371/journal.pgph.0005037.r001

**Authors:** Laston Gonah

23 Sep 2025

PGPH-D-25-02074

The Dichotomy of Progress: Public Perception, Scientific Evidence, and the Future of Global Health Governance—Insights from Georgia

PLOS Global Public Health

Dear Dr./Prof Zurab Alkhanishvil,

Thank you for submitting your manuscript to PLOS Global Public Health. After careful consideration, we feel that it has merit but does not fully meet PLOS Global Public Health’s publication criteria as it currently stands. Therefore, we invite you to submit a revised version of the manuscript that addresses the points raised during the review process.

For the reasons detailed in the reviewer comments (attached below), we cannot accept the manuscript in its current form. However, we invite you to submit a **major revision**  that fully addresses the reviewers’ concerns, questions, and suggestions. If you and your co-authors are able to provide a revised version that responds comprehensively to these points, we would be pleased to reconsider the manuscript for publication.

Please review the comments carefully, paying particular attention to:

- Developing a robust theoretical framework,
- Aligning the research question/objectives with the methodology, methods, results, and discussion, and
- Ensuring clarity, logical flow, and a balanced acknowledgement of the study’s limitations.

Please submit your revised manuscript by 01 October 2025. If you will need more time than this to complete your revisions, please reply to this message or contact the journal office at globalpubhealth@plos.org. When you're ready to submit your revision, log on to https://www.editorialmanager.com/pgph/ and select the 'Submissions Needing Revision' folder to locate your manuscript file.

Please include the following items when submitting your revised manuscript:

- A rebuttal letter that responds to each point raised by the editor and reviewer(s). You should upload this letter as a separate file labeled 'Response to Reviewers'.
- A marked-up copy of your manuscript that highlights changes made to the original version. You should upload this as a separate file labeled 'Revised Manuscript with Track Changes'.
- An unmarked version of your revised paper without tracked changes. You should upload this as a separate file labeled 'Manuscript'.

Guidelines for resubmitting your figure files are available below the reviewer comments at the end of this letter.

We look forward to receiving your revised manuscript.

Kind regards,

Laston Gonah

Academic Editor

PLOS Global Public Health

Journal Requirements:

1. In the ethics statement in the Methods, you have specified that verbal consent was obtained. Please provide additional details regarding how this consent was documented and witnessed, and state whether this was approved by the IRB.

If the reviewer comments include a recommendation to cite specific previously published works, please review and evaluate these publications to determine whether they are relevant and should be cited. There is no requirement to cite these works unless the editor has indicated otherwise.

Additional Editor Comments:

Dear Dr/Prof Zurab Alkhanishvil and co-authors

Thank you for submitting “The Dichotomy of Progress: Public Perception, Scientific Evidence, and the Future of Global Health Governance—Insights from Georgia” to PLOS Global Public Health.

For the reasons detailed in the reviewer comments (attached below), we cannot accept the manuscript in its current form. However, we invite you to submit a major revision that fully addresses the reviewers’ concerns, questions, and suggestions. If you and your co-authors are able to provide a revised version that responds comprehensively to these points, we would be pleased to reconsider the manuscript for publication.

Please review the comments carefully, paying particular attention to:

• Developing a robust theoretical framework,

• Aligning the research question/objectives with the methodology, methods, results, and discussion, and

• Ensuring clarity, logical flow, and a balanced acknowledgement of the study’s limitations.

We look forward to receiving your revised submission.

Sincerely,

Laston Gonah

Academic Editor

PLOS Global Public Health

[Note: HTML markup is below. Please do not edit.]

Reviewers' comments:

Reviewer's Responses to Questions

**Comments to the Author**

1. Does this manuscript meet PLOS Global Public Health’s publication criteria?

Reviewer #1: Partly

Reviewer #2: Yes

**********

2. Has the statistical analysis been performed appropriately and rigorously?

Reviewer #1: I don't know

Reviewer #2: No

**********

3. Have the authors made all data underlying the findings in their manuscript fully available (please refer to the Data Availability Statement at the start of the manuscript PDF file)?

The PLOS Data policy

Reviewer #1: Yes

Reviewer #2: Yes

**********

4. Is the manuscript presented in an intelligible fashion and written in standard English?

Reviewer #1: No

Reviewer #2: No

**********

Reviewer #1: The first line of the abstract should be revised for clarity. While I understand the 'troubling gap' you are trying to illustrate, I think this should be the gap between "objective progress in health outcomes and the public perception of that objective progress".

The words "in a post-Soviet transitional society" are unnecessary unless you're going to provide some context on what significance being post-Soviet and transitional has on public perception on health. If you'd like to retain this next, I would suggest a paragraph or two reviewing the literature on post Soviet countries and providing an operational definition on 'transitional' and what impact that has on public health knowledge/perceptions, or on factors that may influence perceptions. While I understand this has been included in the discussion section - the impact of this is that I feel this article reads in a topsy turvy manner and should be restructured.

It is good that the survey was carried out in both urban and rural settings, however I can't help but feel that a qualitative component, even if just interviewing five participants per setting, would have offered some richness to the statistical findings. In the absence of qualitative data, I would have liked to see more literature review of surveys from Georgia to justify your findings.

Your recommendations are really important, including that there be localized, community-centric health communications. I would love to see this article published, but not in this current iteration.

Reviewer #2: The “progress paradox” concept is not clearly distinguished from existing research on health literacy, misinformation, or public trust, making the novelty of the argument weak.

Lacks a robust theoretical framework; the manuscript reads more like an advocacy essay than a rigorously grounded empirical study.

Survey instrument is not provided; without the exact questionnaire it is impossible to judge the validity of key constructs such as “belief that life expectancy has decreased.”

No details on response rate, handling of nonresponse bias, or justification for the random walk sampling method, which undermines the claim of a nationally representative sample.

Inferential statistics are mentioned (chi-square, t-tests) but p-values, confidence intervals, and effect sizes are missing, weakening the credibility of the analysis.

Findings from Georgia are repeatedly generalized to global health governance; given the country’s unique post-Soviet sociopolitical context, these extrapolations are speculative.

The discussion conflates local findings (e.g., antibiotic misconceptions) with global trends without clear analytical linkage.

Policy recommendations—localized communication, youth empowerment, and digital media—are broad and generic, lacking empirical evidence or case studies to demonstrate their effectiveness.

The historical review of global health governance is lengthy and distracts from the core empirical findings; the manuscript would benefit from significant condensation.

Table 1 is difficult to read and lacks key statistical information such as confidence intervals?

Terminology is inconsistent (for example, “global health governance” vs. “health governance”).

Several references are outdated or only loosely related; the literature review needs updating and tightening.

The limitations section underplays the implications for external validity and does not sufficiently address cultural or linguistic constraints of the Georgian-language survey.

Abstract is lacking the main structural information. Metholodology ? setting?

Overall editing for conciseness and improved flow is needed.

**********

what does this mean?). If published, this will include your full peer review and any attached files.

**Do you want your identity to be public for this peer review?**  If you choose “no”, your identity will remain anonymous but your review may still be made public.

For information about this choice, including consent withdrawal, please see our Privacy Policy

Reviewer #1: No

Reviewer #2: **Yes:** Sumia Andleeb Abbasi

**********

[NOTE: If reviewer comments were submitted as an attachment file, they will be attached to this email and accessible via the submission site. Please log into your account, locate the manuscript record, and check for the action link "View Attachments". If this link does not appear, there are no attachment files.]

# Author comment: Author response to Decision Letter 1

**DOI:** 10.1371/journal.pgph.0005037.r002

26 Jan 2026

Attachment Submitted filename: Response to Reviewers.docx

# Peer review: Decision Letter 1

**DOI:** 10.1371/journal.pgph.0005037.r003

**Authors:** Emma Campbell

29 Apr 2026

PGPH-D-25-02074R1

The Dichotomy of Progress: Public Perceptions of Global Health Trends and Antibiotic Knowledge in Georgia

PLOS Global Public Health

Dear Dr. Alkhanishvili,

Thank you for submitting your manuscript to PLOS Global Public Health. After careful consideration, we feel that it has merit but does not fully meet PLOS Global Public Health’s publication criteria as it currently stands. Therefore, we invite you to submit a revised version of the manuscript that addresses the points raised during the review process.

The revised manuscript has been assessed and the reviewer has requested some final edits to condense some areas of the manuscript and improve overall clarity.

Please submit your revised manuscript by Jun 28 2026 11:59PM. If you will need more time than this to complete your revisions, please reply to this message or contact the journal office at globalpubhealth@plos.org. When you're ready to submit your revision, log on to https://www.editorialmanager.com/pgph/ and select the 'Submissions Needing Revision' folder to locate your manuscript file.

Please include the following items when submitting your revised manuscript:

- A letter that responds to each point raised by the editor and reviewer(s). You should upload this letter as a separate file labeled 'Response to Reviewers'.
- A marked-up copy of your manuscript that highlights changes made to the original version. You should upload this as a separate file labeled 'Revised Manuscript with Track Changes'.
- An unmarked version of your revised paper without tracked changes. You should upload this as a separate file labeled 'Manuscript'.

Guidelines for resubmitting your figure files are available below the reviewer comments at the end of this letter.

As the corresponding author, your ORCID iD is verified in the submission system and will appear in the published article. PLOS supports the use of ORCID, and we encourage all coauthors to register for an ORCID iD and use it as well. Please encourage your coauthors to verify their ORCID iD within the submission system before final acceptance, as unverified ORCID iDs will not appear in the published article. *Only*  the individual author can complete the verification step; PLOS staff *cannot*  verify ORCID iDs on behalf of authors.

We look forward to receiving your revised manuscript.

Kind regards,

Emma Campbell, Ph.D

Staff Editor

PLOS Global Public Health

**Journal Requirements:**

If the reviewer comments include a recommendation to cite specific previously published works, please review and evaluate these publications to determine whether they are relevant and should be cited. There is no requirement to cite these works unless the editor has indicated otherwise.

**Additional Editor Comments (if provided):**

[Note: HTML markup is below. Please do not edit.]

Reviewers' comments:

Reviewer's Responses to Questions

**Comments to the Author**

Reviewer #3: (No Response)

**********

publication criteria?

Reviewer #3: Yes

**********

3. Has the statistical analysis been performed appropriately and rigorously?

Reviewer #3: Yes

**********

4. Have the authors made all data underlying the findings in their manuscript fully available (please refer to the Data Availability Statement at the start of the manuscript PDF file)?

The PLOS Data policy

Reviewer #3: No

**********

5. Is the manuscript presented in an intelligible fashion and written in standard English?

Reviewer #3: Yes

**********

**Reviewer #3:** The manuscript has been improved. My main concern is the length, given that this is a rather limited suervey. I suggest that the introduction, including the genreal introduction, the theoretical foundation, and the aims and objectives should be shortened.

The random walk methods needs to be explained briefly, or a reference given.

**********

what does this mean?). If published, this will include your full peer review and any attached files.

**Do you want your identity to be public for this peer review?**  If you choose “no”, your identity will remain anonymous but your review may still be made public.

For information about this choice, including consent withdrawal, please see our Privacy Policy

Reviewer #3: No

**********

[NOTE: If reviewer comments were submitted as an attachment file, they will be attached to this email and accessible via the submission site. Please log into your account, locate the manuscript record, and check for the action link "View Attachments". If this link does not appear, there are no attachment files.]

**Figure Resubmissions:**

# Author comment: Author response to Decision Letter 2

**DOI:** 10.1371/journal.pgph.0005037.r004

20 May 2026

Attachment Submitted filename: Response to Reviewer .pdf

# Editor report: Decision Letter 2

**DOI:** 10.1371/journal.pgph.0005037.r005

**Authors:** Katherine Demi Kokkinias

24 Jun 2026

PGPH-D-25-02074R2

The Dichotomy of Progress: Public Perceptions of Global Health Trends and Antibiotic Knowledge in Georgia

PLOS Global Public Health

Dear Dr. Alkhanishvili,

Thank you for submitting your manuscript to PLOS Global Public Health. After careful consideration, we feel that it has merit but does not fully meet PLOS Global Public Health’s publication criteria as it currently stands. Therefore, we invite you to submit a revised version of the manuscript that addresses the points raised during the review process.

==============================

There are a few remaining concerns with this manuscript. Please added additional information regarding how the survey instrument was validated. We note that a description of the perception scoring is included as a note beneath table 1. Please also include how the perception items were scored in the Methods section. It would also be beneficial to compare and contract your results to the existing literature on antibiotic knowledge and perceptions in the United States within the introduction and discussion sections.

Could you please revise the manuscript to carefully address the concerns raised?

==============================

Please submit your revised manuscript by Jul 24 2026 11:59PM. If you will need more time than this to complete your revisions, please reply to this message or contact the journal office at globalpubhealth@plos.org. When you're ready to submit your revision, log on to https://www.editorialmanager.com/pgph/ and select the 'Submissions Needing Revision' folder to locate your manuscript file.

Please include the following items when submitting your revised manuscript:

- A letter that responds to each point raised by the editor and reviewer(s). You should upload this letter as a separate file labeled 'Response to Reviewers'.
- A marked-up copy of your manuscript that highlights changes made to the original version. You should upload this as a separate file labeled 'Revised Manuscript with Track Changes'.
- An unmarked version of your revised paper without tracked changes. You should upload this as a separate file labeled 'Manuscript'.

Guidelines for resubmitting your figure files are available below the reviewer comments at the end of this letter.

As the corresponding author, your ORCID iD is verified in the submission system and will appear in the published article. PLOS supports the use of ORCID, and we encourage all coauthors to register for an ORCID iD and use it as well. Please encourage your coauthors to verify their ORCID iD within the submission system before final acceptance, as unverified ORCID iDs will not appear in the published article. *Only*  the individual author can complete the verification step; PLOS staff *cannot*  verify ORCID iDs on behalf of authors.

We look forward to receiving your revised manuscript.

Kind regards,

Katherine Demi Kokkinias, Ph.D.

Staff Editor

PLOS Global Public Health

**Journal Requirements:**

If the reviewer comments include a recommendation to cite specific previously published works, please review and evaluate these publications to determine whether they are relevant and should be cited. There is no requirement to cite these works unless the editor has indicated otherwise.

Please review your reference list to ensure that it is complete and correct. If you have cited papers that have been retracted, please include the rationale for doing so in the manuscript text, or remove these references and replace them with relevant current references. Any changes to the reference list should be mentioned in the rebuttal letter that accompanies your revised manuscript. If you need to cite a retracted article, indicate the article’s retracted status in the References list and also include a citation and full reference for the retraction notice.

**Additional Editor Comments (if provided):**

[Note: HTML markup is below. Please do not edit.]

**Reviewers' comments:**

[NOTE: If reviewer comments were submitted as an attachment file, they will be attached to this email and accessible via the submission site. Please log into your account, locate the manuscript record, and check for the action link "View Attachments". If this link does not appear, there are no attachment files.]

**Figure Resubmissions:**

# Author comment: Author response to Decision Letter 3

**DOI:** 10.1371/journal.pgph.0005037.r006

24 Jul 2026

Attachment Submitted filename: Response_to_Reviewers.docx

# Editor report: Decision Letter 3

**DOI:** 10.1371/journal.pgph.0005037.r007

**Authors:** Julia Robinson

29 Jul 2026

The Dichotomy of Progress: Public Perceptions of Global Health Trends and Antibiotic Knowledge in Georgia

PGPH-D-25-02074R3

Dear Mr. Alkhanishvili,

We are pleased to inform you that your manuscript 'The Dichotomy of Progress: Public Perceptions of Global Health Trends and Antibiotic Knowledge in Georgia' has been provisionally accepted for publication in PLOS Global Public Health.

Before your manuscript can be formally accepted you will need to complete some formatting changes, which you will receive in a follow up email. A member of our team will be in touch with a set of requests.

Please note that your manuscript will not be scheduled for publication until you have made the required changes, so a swift response is appreciated.

IMPORTANT: The editorial review process is now complete. PLOS will only permit corrections to spelling, formatting or significant scientific errors from this point onwards. Requests for major changes, or any which affect the scientific understanding of your work, will cause delays to the publication date of your manuscript.

If your institution or institutions have a press office, please notify them about your upcoming paper to help maximize its impact. If they'll be preparing press materials, please inform our press team as soon as possible -- no later than 48 hours after receiving the formal acceptance. Your manuscript will remain under strict press embargo until 2 pm Eastern Time on the date of publication. For more information, please contact globalpubhealth@plos.org.

Thank you again for supporting Open Access publishing; we are looking forward to publishing your work in PLOS Global Public Health.

Best regards,

Julia Robinson

Executive Editor

PLOS Global Public Health

***********************************************************

Reviewer Comments (if any, and for reference):
